# Descriptor for C_2_N-Supported Single-Cluster Catalysts in Bifunctional Oxygen Evolution and Reduction Reactions

**DOI:** 10.1021/acs.jpclett.3c03573

**Published:** 2024-02-15

**Authors:** Jing Pan, Min Li, Ivo A. W. Filot, Hui Wang, Emiel J. M. Hensen, Long Zhang

**Affiliations:** † School of Physics, Hunan Key Laboratory of Super Microstructure and Ultrafast Process, State Key Laboratory of Powder Metallurgy, Central South University, Changsha 410083, China; ‡ Department of Chemical Engineering and Chemistry, Eindhoven University of Technology, P.O. Box 513, 5600 MB Eindhoven, The Netherlands

## Abstract

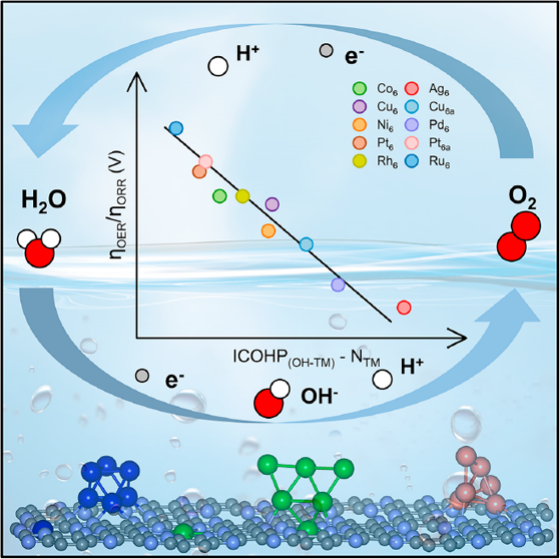

Developing highly active cluster catalysts for the bifunctional oxygen evolution reaction (OER) and oxygen reduction reaction (ORR) is significant for future renewable energy technology. Here, we employ first-principles calculations combined with a genetic algorithm to explore the activity trends of transition metal clusters supported on C_2_N. Our results indicate that the supported clusters, as bifunctional catalysts for the OER and the ORR, may outperform single-atom catalysts. In particular, the C_2_N-supported Ag_6_ cluster exhibits outstanding bifunctional activity with low overpotentials. Mechanistic analysis indicates that the activity of the cluster is related to the number of atoms in the active site as well as the interaction between the intermediate and the cluster. Accordingly, we identify a descriptor that links the intrinsic properties of the clusters with the activity of both the OER and the ORR. This work provides guidelines and strategies for the rational design of highly efficient bifunctional cluster catalysts.

The development of new technologies for energy conversion is crucial to addressing the energy crisis. In particular, energy technologies based on renewable energy sources (e.g., solar, water, and air) are regarded as promising ways to develop clean energy-related devices. Catalysts are the core of these devices, in which a series of electrochemical redox reactions take place on the cathode and anode. For example, the oxygen evolution reaction (OER) serves as the anode reaction in a water-splitting electrolyzer, while the oxygen reduction reaction (ORR) is the cathode reaction occurring at the electrodes of electrolysis cells.^1,2^ There is growing interest in the study of the OER and ORR, as these two reactions play important roles in renewable energy technologies. A bifunctional catalyst for the OER and ORR could be employed in a unitized regenerative fuel cell, coupling with intermittent energy sources (e.g., wind and solar energies) to efficiently shift electricity to the grid during peak demand.^3^ However, the sluggish kinetics during the OER and ORR hinder the development of highly efficient energy conversion devices.^4,5^ Hence, the design of high-performance catalysts to overcome the kinetic barrier of the reaction is greatly significant.

To date, platinum group metals (PGMs), such as Pt, Ir, and Ru, are commonly adopted as state-of-the-art electrocatalysts for the OER and ORR.^6−8^ However, the high cost and scarcity of PGMs impose restrictions on their large-scale and sustainable utilization. One of the strategies for solving this problem is to highly disperse the PGMs on the support and form single-atom catalysts (SACs)^9−11^ or single-cluster catalysts (SCCs),^12,13^ which recently have been demonstrated as potential substitutes for PGM-based electrocatalysts.^14−18^ To develop the highly efficient SACs for the OER and ORR, extensive trial-and-error approaches or high-throughput density functional theory (DFT) calculations have been performed in recent studies.^19−22^ Theoretical models have been constructed to correlate the catalytic activity of the OER and ORR with the properties of SACs, such as the d-band center,^17^ electronic spin moment,^23^ and adsorption energy of reaction species.^24^ The descriptors for SACs in the OER and ORR have been well established in previous works.^25−28^

Similar to SACs, SCCs provide an alternative way to efficiently utilize transition metal (TM) atoms in various catalytic processes.^29−32^ Some of the remarkable advantages of cluster catalysts are their special structural and electronic properties.^33−35^ The exposed atoms of clusters with a suitable ensemble size can serve as a mutisite active center during catalysis, which has become very attractive in a wide range of applications in the field of heterogeneous catalysis.^36−39^ However, previous reports often represent case studies, such as computational investigation of specific clusters, like Fe_3_^34^ and W_4_.^40^ Other theoretical studies focus on computational screening of precious trimetallic clusters supported on N-doped graphene for the OER and ORR.^41^ Recently, a C_2_N monolayer has been successfully synthesized in the laboratory,^42^ which has an inherently porous structure enabling stable anchoring of clusters. In particular, six pyridinic N atoms in the hollow site of C_2_N could strongly interact with metal clusters, thereby favoring the synthesis of subnanometer cluster catalysts. Therefore, C_2_N-supported cluster catalysts were considered as promising catalytic systems.^43,44^ While certain efforts have been made to identify high-performance three-atom and four-atom cluster catalysts for various reactions,^45−48^ the development of stable and active clusters for the bifunctional OER and ORR is still far from satisfactory. Moreover, the increase in the number of atoms in clusters dramatically increases the complexity of the catalyst and the diversity of the reaction sites, which poses a challenge for determining their structure and catalytic properties. Due to the complex atomic environment of cluster catalysts, the descriptors used for SACs, such as the metal–oxygen bond order,^49^ d-band center,^50^ and electronic spin moment,^23^ may not be applicable for evaluating the catalytic activity of SCCs. Therefore, resolving the structure of SCCs and correlating the intrinsic properties of the active center with the catalytic activity of supported clusters are vital for the rational design of highly efficient bifunctional cluster catalysts.

Here, we employ a method rooted in a first-principles-based genetic algorithm (GA) to resolve C_2_N-supported SCCs. Given that six-atom clusters strike a balance between computational feasibility and capturing essential catalytic properties, we determine the structure of eight distinct TM_6_ (TM = Co, Ni, Cu, Ru, Rh, Pd, Ag, or Pt) clusters supported on C_2_N for trend studies. Then, we explore the free energy diagrams for the OER and ORR on the supported clusters, followed by identifying the potential-determining step (PDS) and evaluating the overpotentials. With the help of the established scaling relations for the adsorption free energies of intermediates, the catalytic activity trends in terms of the overpotential can be determined and a strategy for improving the catalytic activity of OER and ORR is proposed. We find that the Ag_6_ cluster supported on C_2_N has the lowest bifunctional OER and ORR overpotentials among the SCCs and SACs. A mechanistic study shows that the activity of the clusters is related to the local atomic environment of the active center and the interaction between the intermediate and the cluster. We further identify a descriptor for predicting the OER and ORR activity of the supported clusters. This work builds a picture of the bifunctional activity of supported clusters and establishes the structure–activity relationship for the rational design of SCCs.

Resolving the structure of a supported cluster catalyst is crucial to unraveling the nature of its active sites and catalytic properties. We determined the stable and metastable structure of clusters on a C_2_N monolayer by employing a structure evolutionary method based on a GA and first-principles approach. In this computational framework, DFT calculations have been performed, mostly determining the total energy and characteristics of the optimized clusters. The computational details for the DFT calculations can be found in the Supporting Information. The structure search begins with an initial population of 12 randomly generated structures. To maintain the diversity of the structures, the initial structures are generated by employing the second-order bond length distribution method, which has exhibited good performance for the random generation of metal clusters.^51^ We considered three typical operations (e.g., crossover, mutation, and selection) in the evolutionary process to iteratively update the population pool. To simulate the natural selection process, two structures in the population are selected as parents to reproduce offspring when their fitness values exceed a random number threshold. The new candidate structures are obtained by repeating the three operations. Typically, the stable structure of TM_6_@C_2_N can be determined within 30 iterations and <360 structure optimizations during the DFT-based GA search process. After convergence of the process, we can identify the optimized structure with the lowest total energy among the candidates, which is determined to be the most stable structure. Using this first-principles-based GA method, we successfully predicted the most stable structure of a ceria-supported gold nanocluster, as reported in our previous study. A detailed description of this method can be found in our previous work.^52^

Figure 1 displays the most stable and metastable structures of TM_6_@C_2_N. To assess the stability of TM_6_@C_2_N, we calculated the adsorption energy and cohesive energy of TM_6_ clusters (Figure S1 and Table S1). The significantly negative values obtained for both adsorption and cohesive energies indicate a strong interaction between the TM_6_ clusters and C_2_N. Consequently, the TM_6_ clusters can be firmly anchored to the support, exhibiting resistance to cluster aggregation under reaction conditions. We observe that Co_6_ and Ru_6_ clusters on C_2_N exhibit bilayer structures with similar morphologies in which three transition metal (TM) atoms bind to the nitrogen atoms of the support. For Ag_6_@C_2_N, we find that Ag atoms are anchored on C_2_N with a two-dimensional (2D) arrangement, while there are four TM atoms directly interacting with the support in the C_2_N-supported Ni_6_, Cu_6_, Pd_6_, Rh_6_, and Pt_6_ clusters. The diverse intrinsic properties of metals and their interactions with the support lead to distinct local coordination environments and various morphologies for the supported clusters. As a result, the catalytic properties of the supported clusters are different. Given the metastable clusters could also play a major role in catalytic reactivity,^53,54^ consideration of the global minimum structure alone may underestimate the activity of the catalysts. In our study, we consider the metastable isomers that have a total energy that is <0.20 eV greater than that of the most stable structure, as the total energies of metastable Cu_6a_@C_2_N and Pt_6a_@C_2_N closely approximate those of their respective most stable counterparts, resulting in small relative energies for Cu_6a_@C_2_N (0.15 eV) and Pt_6a_@C_2_N (0.06 eV). To further confirm the thermodynamic stability of these clusters, we conducted *ab initio* molecular dynamics (AIMD) simulations of the representative stable clusters and their metastable isomers (Pt_6_@C_2_N, Pt_6a_@C_2_N, Cu_6_@C_2_N, and Cu_6a_@C_2_N) at a reaction temperature of 300 K. As displayed in Figure S2, these cluster catalysts can sustain geometrical integrity and exhibit high thermal stability, suggesting the anchored metal clusters on C_2_N could resist aggregation and avoid the formation of large metal particles at the reaction temperature. Therefore, it is necessary to explore the catalytic properties of these two metastable structures in this work.

**Figure 1:**
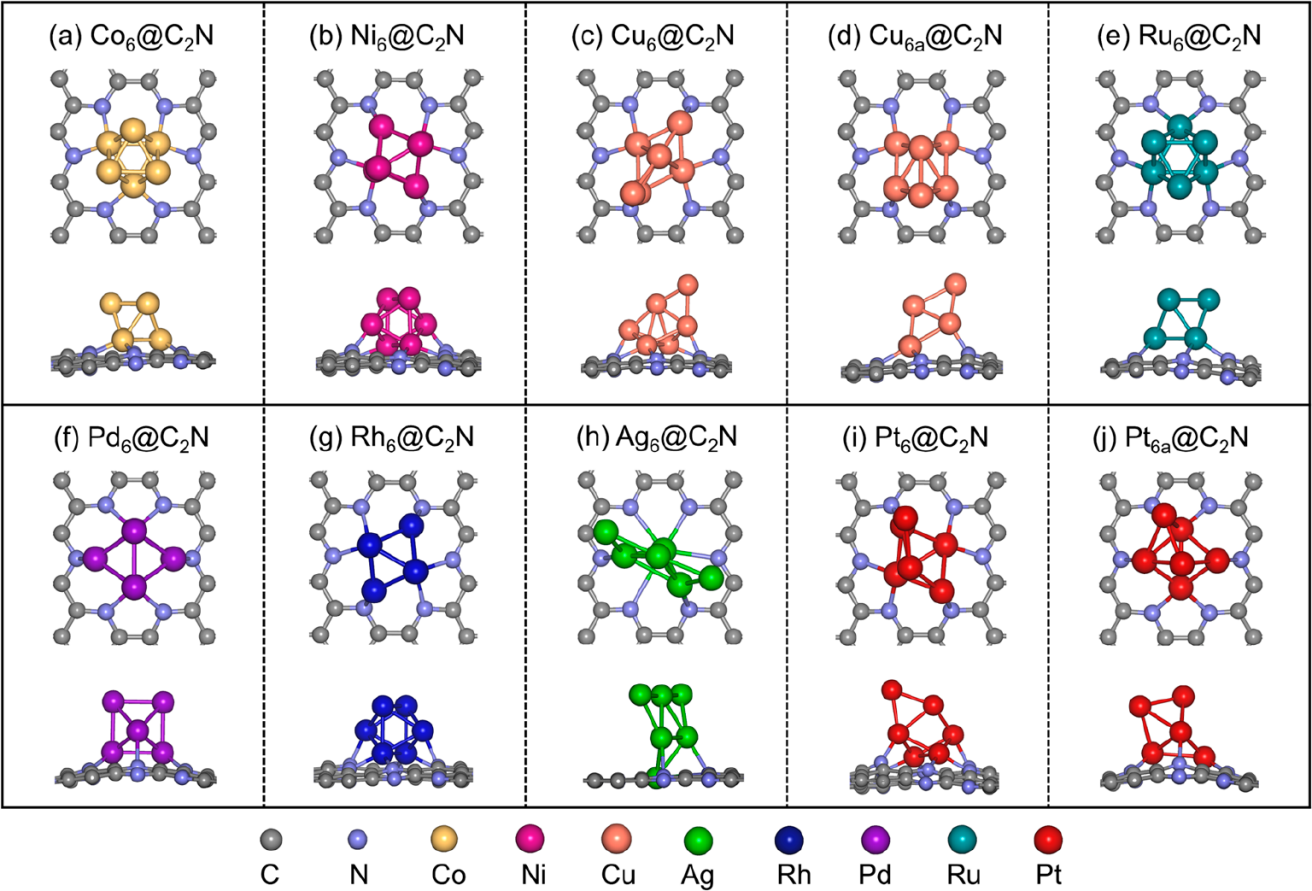
Structures of TM_6_ clusters on C_2_N (structures optimized by GA+DFT): (a) Co_6_@C_2_N, (b) Ni_6_@C_2_N, (c) Cu_6_@C_2_N, (d) Cu_6a_@C_2_N (metastable Cu cluster), (e) Ru_6_@C_2_N, (f) Pd_6_@C_2_N, (g) Rh_6_@C_2_N, (h) Ag_6_@C_2_N, (i) Pt_6_@C_2_N, and (j) Pt_6a_@C_2_N (metastable Pt cluster).

We next explore the typical reaction mechanisms for the OER and ORR on the cluster catalysts. The potential clusters for the bifunctional reactions are screened on the basis of the OER and ORR overpotentials (η_OER_ and η_ORR_, respectively). To identify the adsorption site for the intermediate, we conducted a comparative analysis of the adsorption free energies of OH on various typical sites, as illustrated in Figure S3 and detailed in Table S2. To determine the PDS and obtain the overpotentials, we calculated the reaction free energy for the OER and ORR on TM_6_@C_2_N, as displayed in Figure 2 and Figure S4. For the OER on C_2_N-supported Co_6_, Pt_6_, and Pt_6a_, the PDS with the highest reaction free energy lies in the last step (*OOH → O_2_) with free energy changes (Δ*G*) of 2.39, 2.57, and 2.65 eV, respectively. The high Δ*G* values indicate high OER overpotentials for the catalysts, as shown in Figure 2a, while the third step (*O → *OOH) is the PDS for the OER on Ni_6_@C_2_N, Rh_6_@C_2_N, Ag_6_@C_2_N, Pd_6_@C_2_N, Cu_6_@C_2_N, Cu_6a_@C_2_N, and Ru_6_@C_2_N, with Δ*G* values of 2.37, 2.42, 1.79, 1.96, 2.45, 2.26, and 2.92 eV, respectively. The OER overpotentials for Ni_6_@C_2_N, Rh_6_@C_2_N, Ag_6_@C_2_N, Pd_6_@C_2_N, Cu_6_@C_2_N, Cu_6a_@C_2_N, and Ru_6_@C_2_N are 1.14, 1.19, 0.56, 0.73, 1.22, 1.04, and 1.69 V, respectively. Clearly, Ag_6_@C_2_N exhibits the lowest OER overpotentials among the TM_6_@C_2_N group. This OER overpotential is comparable to the IrO_2_(110) benchmark (0.56 V).^55^

**Figure 2:**
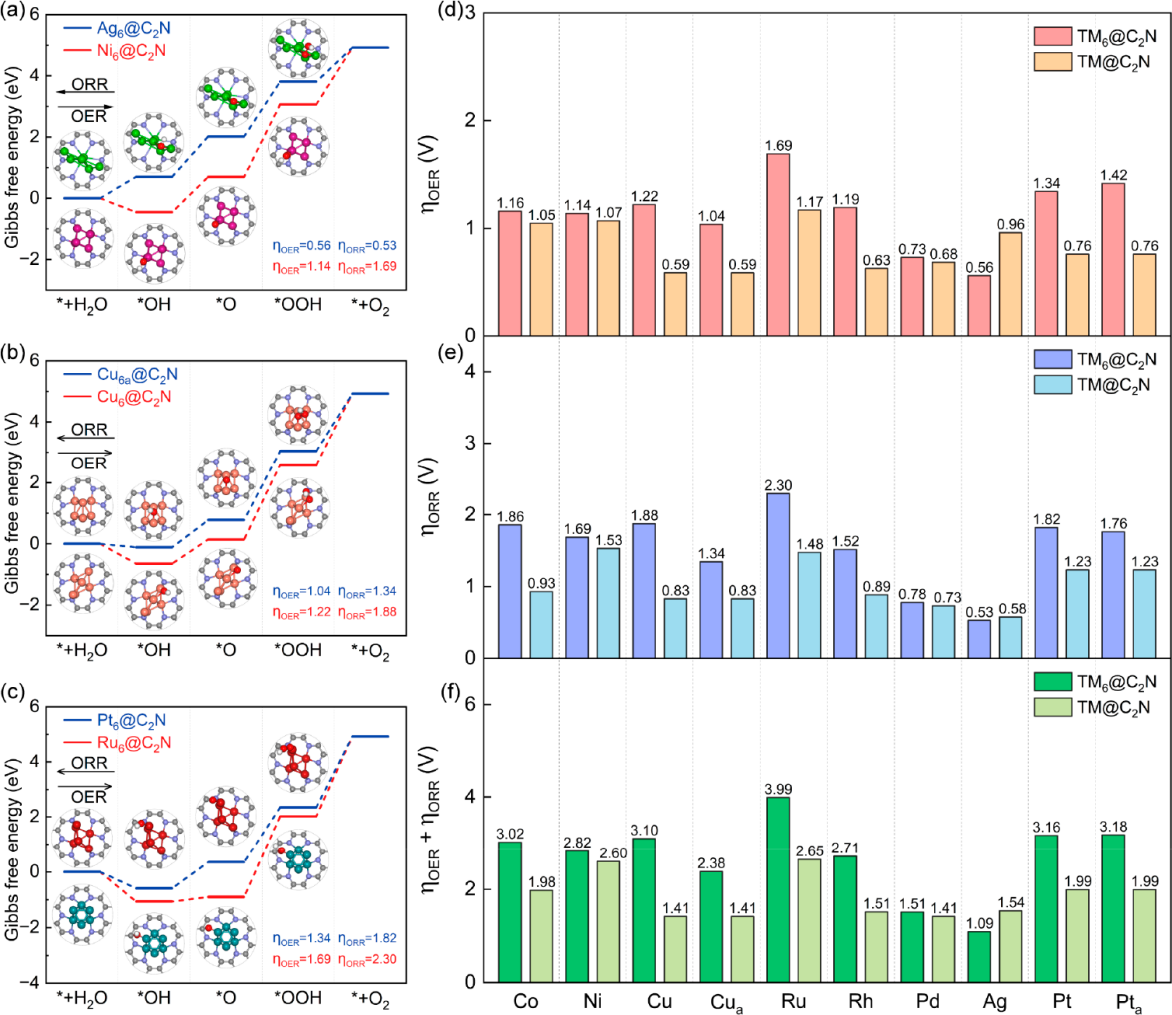
Free energy diagrams of the ORR and OER on (a) Ag_6_@C_2_N and Ni_6_@C_2_N, (b) Cu_6a_@C_2_N and Cu_6_@C_2_N, and (c) Pt_6_@C_2_N and Ru_6_@C_2_N. (d) Overpotentials of the OER for TM_6_@C_2_N and TM@C_2_N, (e) overpotentials of the ORR for TM_6_@C_2_N and TM@C_2_N, and (f) sum of the OER and ORR overpotentials for TM_6_@C_2_N and TM@C_2_N.

Previous studies demonstrated that the adsorption strength of *OH, *O, and *OOH determines the kinetics of the OER and the PDS distribution.^17,56^ To understand the relationship between the adsorption strength of intermediates and the catalytic activity of the clusters, we explore the correlations among the adsorption free energies. A linear scaling relationship between ΔG_*OH_ and ΔG_*O_ is found, as displayed in Figure 3a. Additionally, ΔG_*OOH_ can be strongly correlated to ΔG_*OH_ (Figure 3b), which can be expressed as ΔG_*OOH_ = 0.95ΔG_*OH_ + 3.06 with a high coefficient of determination (*R*^2^ = 0.85). These correlations imply that the clusters with a strong adsorption strength of *OH tend to exhibit strong binding to *O and *OOH. Using the linear scaling relations described above, we further explore the catalytic activity trends by constructing a 2D volcano map for the OER on TM_6_@C_2_N (Figure 3c). This map suggests that a high-performance cluster for the OER should have relatively high ΔG_*OH_ and ΔG_*O_ values. It also indicates that the distribution of the OER activity is subject to the strong linear relationship between ΔG_*OH_ and ΔG_*O_. This is further confirmed by the strong correlation between ΔG_*OH_ (ΔG_*O_) and η_OER_ (Figure 3d and Figure S5a). From the correlations, we observe that the overpotentials for the OER on TM_6_@C_2_N decrease with an increase in the adsorption free energies of the reaction intermediates. As a result, clusters that exhibit relatively weak adsorption of reaction intermediates are more active than those with strong adsorption of reaction intermediates.

**Figure 3:**
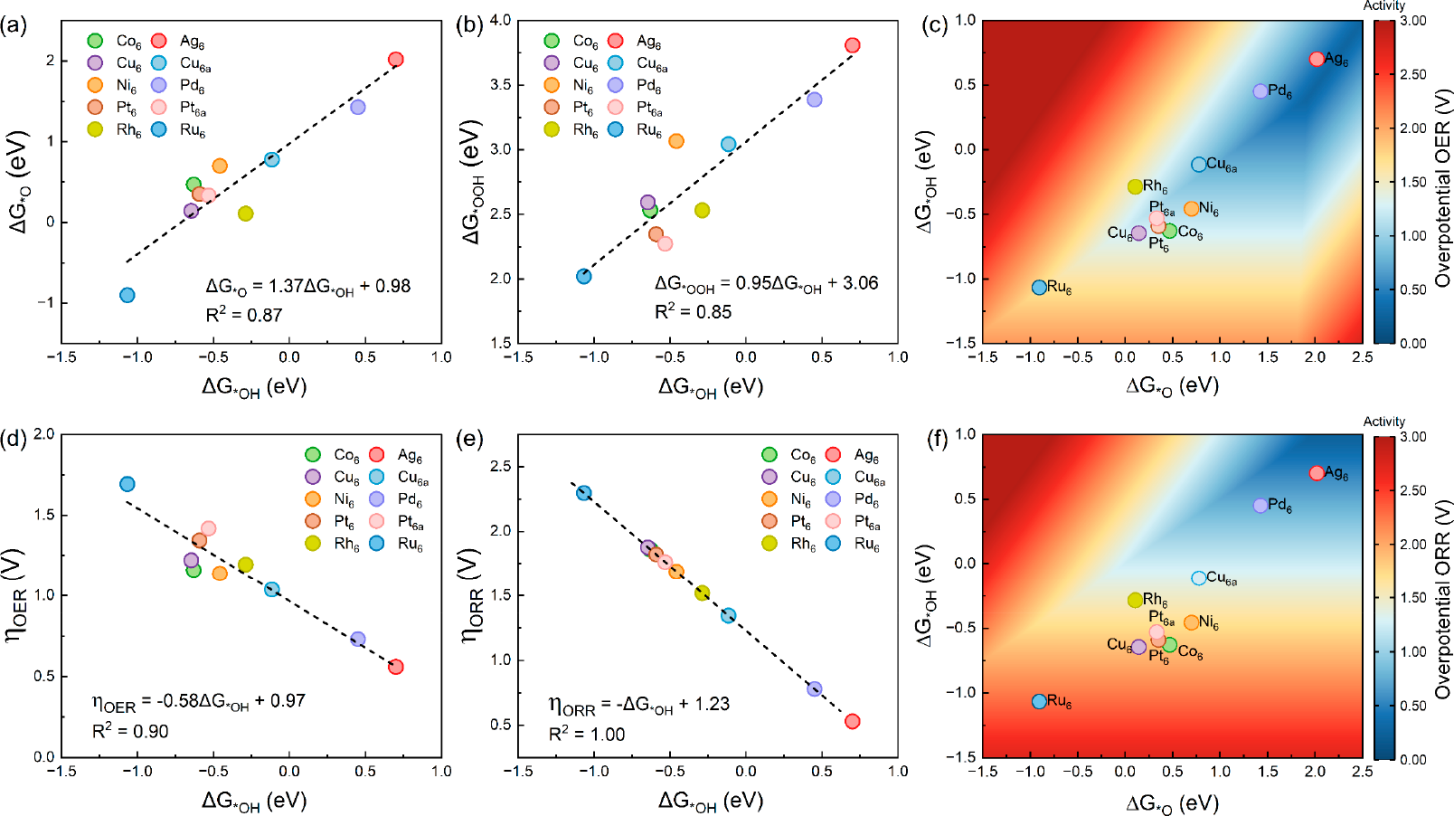
Scaling relationship between the adsorption free energies of intermediates: (a) ΔG_*OH_ vs ΔG_*O_ and (b) ΔG_*OH_ vs ΔG_*OOH_. (c) 2D heat map of overpotentials of the OER on TM_6_@C_2_N. (d) Correlation between the adsorption free energies and overpotentials of the OER. (e) Correlation between the adsorption free energies and overpotentials of the ORR. (f) 2D heat map of the overpotentials of the ORR on TM_6_@C_2_N.

On the basis of the free energy diagrams, we further evaluated the catalytic ORR activity of the clusters. The strong correlation between η_ORR_ and ΔG_*OH_ indicates the second water formation is the PDS for the ORR on all of the TM_6_@C_2_N catalysts (Figure 3e). Additionally, the reaction free energies of the PDS for the ORR on Ag_6_@C_2_N and Pd_6_@C_2_N are both negative values, resulting in relatively low ORR overpotentials that are close to that of Pt(111) (0.45 V).^57^ While the reaction free energy of the PDS is endothermic for the ORR on the remaining clusters, consequently, the ORR overpotentials are much higher than those for Ag_6_@C_2_N and Pd_6_@C_2_N. This is clearly reflected by the 2D volcano map for the ORR on TM_6_@C_2_N, which also indicates that η_ORR_ decreases with a decrease in the adsorption strength of reaction intermediates (Figure 3e and Figure S5b). Considering the two- and four-electron ORR pathways toward different products (i.e., H_2_O_2_ and H_2_O), we can evaluate the selectivity by calculating the adsorption free energy of O (ΔG_*O_). If ΔG_*O_ < Δ*G*(H_2_O_2_) – Δ*G*(H_2_O), namely, ΔG_*O_ < 3.52 eV,^19^ the ORR produces water via the four-electron reaction pathway. Clearly, the adsorption free energy for O species that adsorb on all of the clusters is much lower than 3.52 eV (Table S3). As a result, the final product of the ORR is water for all of the TM_6_@C_2_N catalysts. Therefore, we mainly discuss the four-electron ORR pathway in our study.

It is worth noting that the OER overpotential (1.04 V) of metastable Cu_6a_@C_2_N is lower than that for the most stable Cu_6_@C_2_N (1.22 V). In addition, the ORR catalytic activity (η_ORR_ = 1.34 V) of the metastable Cu cluster is lower than that of the stable one (η_ORR_ = 1.88 V). We further employ the sum of the overpotentials of the OER and ORR (η_sum_ = η_OER_ + η_ORR_) to assess the bifunctional activity, as shown in Figure 2f. The value of η_sum_ ranges from 1.09 to 3.99 V. Among these clusters, Ru_6_@C_2_N exhibits the highest η_sum_ (3.99 V), which is not active for either the OER or the ORR. On the contrary, Ag_6_@C_2_N has the lowest η_sum_, which is identified as a promising cluster catalyst for the bifunctional OER and ORR.

To understand the catalytic difference between cluster catalysts and single-atom catalysts, we further explore the free energy diagrams and determine the overpotentials of the OER and ORR on C_2_N-supported SACs (Figure 2 and Figure S6). Clearly, the catalytic activity trends for the OER and ORR on SACs are different from those on SCCs. For the OER on SACs, Ru@C_2_N exhibits the highest OER overpotential with a value of 1.17 V, while Cu@C_2_N possesses the lowest OER overpotential with a value of 0.59 V, which is still higher than that of Ag_6_@C_2_N (0.56 V). In other words, the cluster catalyst could perform better in the OER than the SACs could. On the contrary, the ORR overpotentials of Ag@C_2_N (0.58 V) are also higher than that of Ag_6_@C_2_N (0.53 V). The rest of the SACs exhibit lower ORR potentials compared to those of the corresponding TM_6_@C_2_N catalysts. The correlation between η_OER_ and the adsorption free energy is weak for the OER on SACs (Figure S7a–c). Notably, the data point of Ag@C_2_N seems to deviate from the expected linear relationship between η_ORR_ and the adsorption free energy of the intermediates (Figure S7d–f). This is attributed to the distinct distribution of the PDS for the ORR on SACs. We then evaluate the activity of C_2_N-supported SACs for the bifunctional OER and ORR. The Ru@C_2_N has the highest η_sum_ (2.65 V), while Cu@C_2_N and Pd@C_2_N both exhibit the lowest η_sum_ (1.41 V) for the bifunctional OER and ORR among the SACs. However, the η_sum_ values of these two SACs are still much higher than that of Ag_6_@C_2_N (η_sum_ = 1.09 V), indicating that the supported cluster catalysts for the bifunctional OER and ORR could be more advantageous than the SACs. Furthermore, we employ AIMD simulations to confirm that Ag_6_@C_2_N is thermodynamically stable at the reaction temperature (Figure S8).

The results presented above show that the adsorption strength of intermediates strongly correlates with the overpotentials of the OER and ORR. To reveal the underlying catalytic activity trends and the role of different metal clusters, we conducted electronic structure analysis of these SCCs. Figure 4 and Figure S9 show the projected density of states (PDOS) on the adsorption site of TM_6_@C_2_N. We observe that the distribution of TM d states of the clusters is dependent on the specific TM atom (Figure 4). The adsorption site of the Ag cluster possesses fewer d states near the Fermi level compared to other TM clusters, as reflected by its relatively low d-band center. Consequently, the supported Ag cluster exhibits weak back-donation to the antibonding orbitals of the O 2p orbitals. Additionally, the almost fully occupied Ag 4d orbitals further hinder the donation of an electron from the O orbitals, resulting in a weak interaction between the adsorbed OH and the Ag cluster. This is supported by the distribution of O 2p states of the OH and the d or sp orbitals of TM clusters (Figure 4 and Figure S10). The overlap between the O 2p states and the Ag 4d states is less pronounced and localized than the overlap between the O 2p states and those of other TM d states. A similar trend of interaction between the sp states of TM clusters and the O 2p states is observed. In other words, the sp states of the clusters also contribute to the adsorption strength of intermediates. This explains why only the d-band center could not reproduce the trend of the OH adsorption free energy.

**Figure 4:**
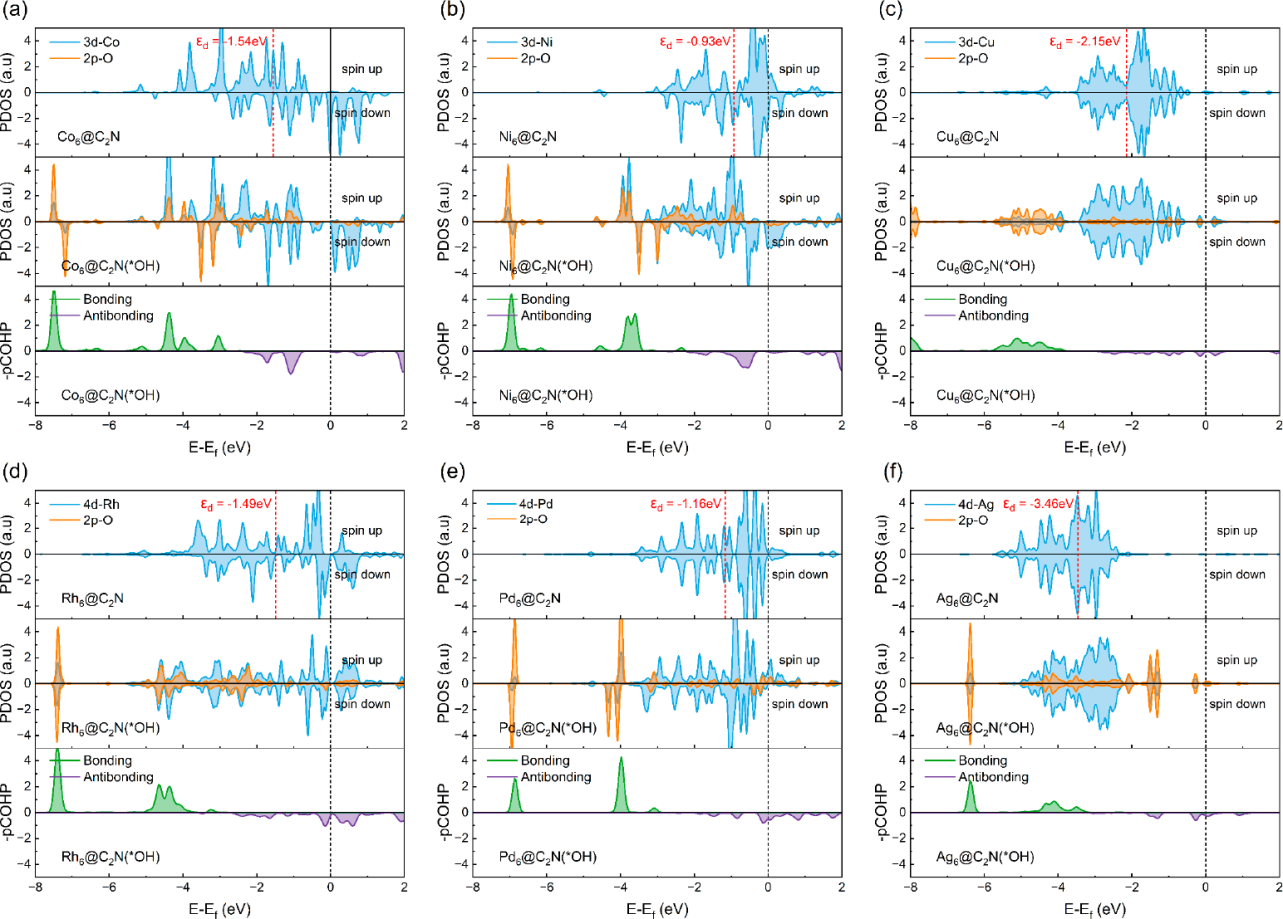
Projected density of states (PDOS) for pristine and OH-adsorbed clusters as well as projected crystal orbital Hamilton populations (pCOHPs) for OH adsorbed on (a) Co_6_@C_2_N, (b) Ni_6_@C_2_N, (c) Cu_6_@C_2_N, (d) Rh_6_@C_2_N, (e) Pd_6_@C_2_N, and (f) Ag_6_@C_2_N. The Fermi energy level is set to 0 eV.

We further conducted a projection of the crystal orbital Hamilton population (pCOHP) to analyze the interaction between the cluster and the intermediate. In the pCOHP analysis of OH-TM in OH-adsorbed TM_6_@C_2_N, we observe antibonding orbital populations below the Fermi level for all cases. Furthermore, the bonding orbital populations of the Ag and Pd clusters are smaller than those for other TM clusters. This is supported by the relatively higher average integrated crystal orbital Hamilton populations (ICOHPs) for OH-Ag and OH-Pd, with values of −1.33 and −1.57, respectively. Here, the ICOHP is calculated on the basis of the interaction between the reaction intermediate and TM atoms in the adsorption site. This explains the weak adsorption of OH on the Ag and Pd clusters, while the bonding orbital populations of the Co and Rh clusters below the Fermi level are more pronounced than those of other clusters, leading to relatively lower ICOHPs for OH-Co (−3.00) and OH-Rh (−2.91). Consequently, the adsorption of OH on Co and Rh clusters is apparently stronger than that on other clusters. The strong adsorption of OH on the cluster inevitably increases the reaction free energy of the PDS, consequently increasing the overpotentials. On the contrary, the second water formation is the PDS for the ORR on all of the clusters (Figure 2 and Figure S4). The enhanced OH adsorption also increases the reaction free energy for water formation. Thus, a weaker OH adsorption results in a lower ORR overpotential.

To achieve a rational design of highly efficient cluster catalysts, it is crucial to identify the characteristics of the active site. Recently, several theoretical studies reported that the catalytic activity of single-atom catalysts or double-atom catalysts can be linked with the adsorption energy of reaction intermediates.^58−60^ Nevertheless, a descriptor for evaluating the bifunctional OER and ORR activities of cluster catalysts is absent. In this study, we find that the adsorption free energies of intermediates strongly correlate with the OER and ORR overpotentials. However, the intrinsic properties of the cluster catalysts that govern activity remain obscured. Furthermore, engineering the active center to tune the adsorption strength for optimal activity via the adsorption–activity relationship is impractical without understanding the intrinsic links between the properties of the cluster catalysts and their activity. Therefore, it is important to determine the properties of the cluster catalysts with explicit physical meanings that are strongly correlated to their activity. Considering the fact that the interaction between the intermediate and cluster may determine the catalytic reactivity of clusters, we first plot the overpotentials versus the ICOHPs of O-TM, OH-TM, and OOH-TM, as shown in Figure S11 and Table S4. However, the correlations between the overpotentials and ICOHP are not strong, as reflected by the low coefficient of determination. This suggests that ICOHPs alone cannot strongly correlate with the OER and ORR overpotentials for the supported cluster catalysts. Given that the local atomic environment of the active center impacts the catalytic reactivity of the clusters, we introduce the TM atom number in the adsorption site (*N*_TM_). It is known that *N*_TM_ influences the adsorption behavior of the intermediates. An active center with more TM atoms, namely, more binding sites, is more likely to exhibit stronger interaction with the adsorbate. As a result, a cluster with a large *N*_TM_ exhibits stronger adsorption compared to that of a cluster with a small *N*_TM_. Taking into account both *N*_TM_ and the interactions between intermediates and the cluster, we proceed to refine the descriptor as follows: λ = ICOHP_(OH-TM)_ – *N*_TM_. Clearly, we observe a strong correlation between λ and the overpotentials of both the OER and the ORR. The high *R*^2^ values of the correlations show the good performance of the descriptor for the screening of cluster catalysts (Figure 5). In addition, this descriptor links the intrinsic properties of the clusters with their bifunctional activity. No similar trend was observed for the OER and ORR on SACs (Figure S12).

**Figure 5:**
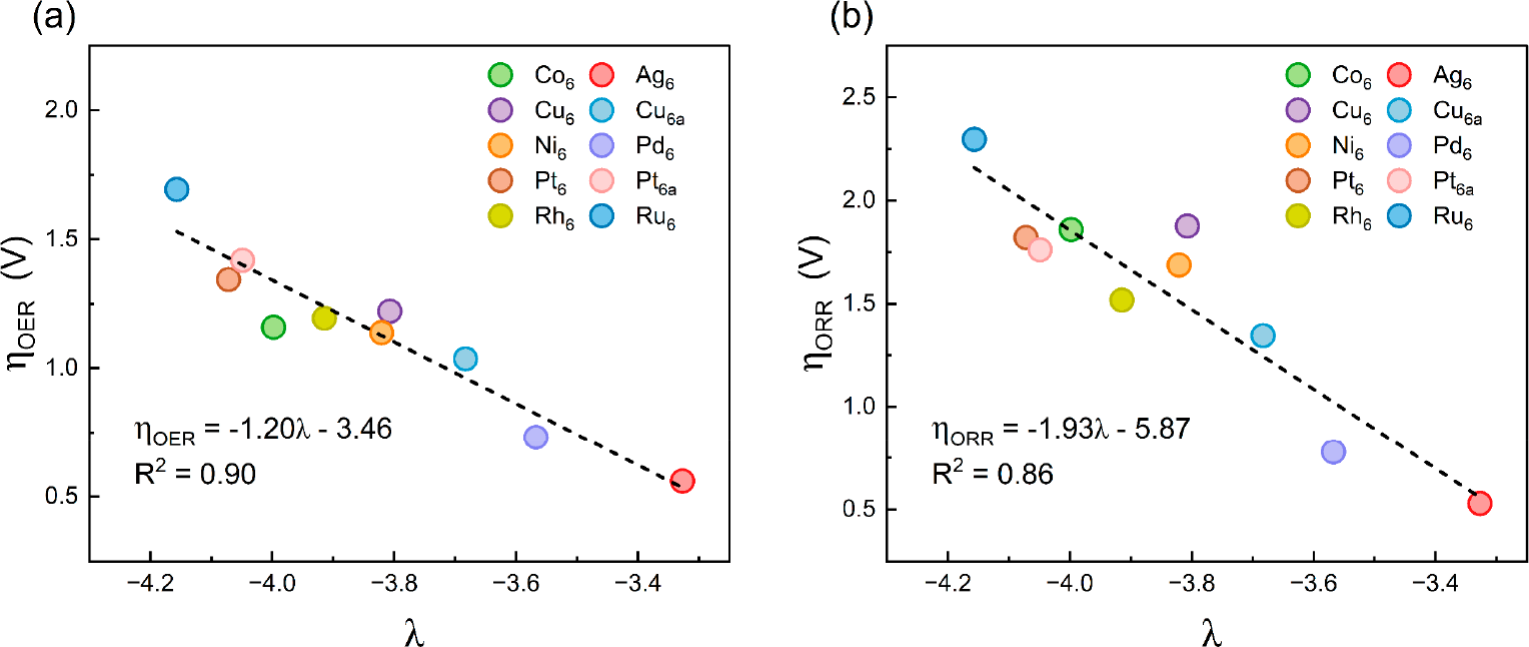
Correlation between the descriptor [λ = ICOHP_(OH-TM)_ – *N*_TM_] and the overpotentials of (a) the OER and (b) the ORR.

In summary, by means of spin-polarized DFT calculations combined with a GA, we determined the structure of TM clusters supported on the experimentally available C_2_N monolayer. We conducted extensive DFT calculations to evaluate the OER and ORR activity of both SCCs and SACs. Our calculations show that the Ag_6_ cluster anchored on C_2_N possesses outstanding bifunctional activity with small OER and ORR overpotentials and demonstrate that the SCCs as bifunctional catalysts could outperform the SACs. Additionally, the high thermal stability of the catalyst is confirmed by AIMD simulations. We further established the catalytic activity trends of the OER and ORR on the clusters on the basis of the intermediate’s adsorption free energies. Our findings suggest that reducing the intermediate adsorption strength could improve the bifunctional OER and ORR activity. Furthermore, the overpotentials for the reaction on SCCs are correlated to the TM atom numbers in the adsorption site and the interaction between the intermediate and the clusters. Importantly, we propose a descriptor that links the local atomic environment and electronic structure of the cluster with the activity of both the OER and the ORR. Our work provides an effective strategy for improving the activity of cluster systems and helps pave the way for the screening and design of efficient bifunctional cluster catalysts.

## Supporting Information Available

Computational methods, detailed structures of C_2_N, TM@C_2_N, and intermediate-adsorbed TM_6_@C_2_N, free energy diagrams of the ORR and OER on clusters, correlations between the adsorption free energies of intermediates, correlations between the overpotentials of SACs and the adsorption free energies of intermediates, PDOS for pristine and OH-adsorbed clusters and pCOHP for OH-adsorbed clusters, and correlations between overpotentials with ICOHP and ICOHP_(OH-TM)_ – *N*_TM_ (PDF)
