# Supplementary material for: Descriptor for C2N-Supported Single-Cluster Catalysts in Bifunctional Oxygen Evolution and Reduction Reactions
Source: J Phys Chem Lett. 2024 Feb 15;15(7):2066–74. doi: 10.1021/acs.jpclett.3c03573 (PMC10895691; doi:10.1021/acs.jpclett.3c03573)
Supplement: Supplementary file 1 — jz3c03573_si_001.pdf [file jz3c03573_si_001.pdf]

Supporting Information

Descriptor for C_2N Supported Single-Cluster Catalysts in Bifunctional Oxygen Evolution and Reduction Reactions

Jing Pan[1], Min Li[1], Ivo A. W. Filot [2], Hui Wang[1],
Emiel J. M. Hensen[2,*], and Long Zhang[1, 2,*]

1 School of Physics, Hunan Key Laboratory of Super Microstructure and Ultrafast Process, State Key Laboratory of Powder Metallurgy, Central South University, Changsha 410083, China

2 Department of Chemical Engineering and Chemistry, Eindhoven University of Technology, P.O. Box 513, 5600 MB Eindhoven, The Netherlands

*E-mail: L.zhang@csu.edu.cn, E.J.M.Hensen@tue.nl

COMPUTATIONAL METHODS

We performed spin-polarized DFT calculations employing the projector-augmented wave (PAW)[1] approach as implemented in VASP 5.4.4.[2,3] We adopted the Perdew-Burke-Ernzerhof (PBE)[4] functional with generalized gradient approximation (GGA).[5] A cut-off energy of 400 eV for constructing valence electron density in the plane-wave basis are used in our calculations. The structure optimization was stopped when Hellmann−Feynman forces acting on atoms were less than 0.02 eV/Å. For the C_2N monolayer, we constructed a 4 × 4 unit cell with an vacuum thickness of 15 Å (Figure S13). Our test shows that the unit cell is large enough to avoid the interaction between periodic images along z direction. Because the constructed unit cells were large enough, we considered a Monkhorst-Pack k-point of 1 × 1 × 1 for the Brillouin zone integration. To model C_2N supported cluster catalysts, a series of TM_6 (TM = Co, Ni, Cu, Ru, Rh, Pd, Ag, and Pt) clusters placed on C_2N (TM_6@C_2N) were considered in our study. Based on our tests for representative cluster catalysts (e.g., Ag_6@C_2N and Pd_6@C_2N), the adsorption free energy of the crucial intermediate (*OH) appears to remain unaffected by the effective U value (Figure S14). Thus, the DFT+U method was not adopted, instead, we employ standard DFT calculations in our study. We employed DFT-based genetic algorithm (DFT+GA) to search the global energy minimum structure of TM clusters on the support. The predicted structure with the lowest total energy among the candidates was determined as the most stable structure of TM_6@C_2N. To explore the thermodynamic stability of the TM_6@C_2N, *Ab initio* molecular dynamics (AIMD) simulations were conducted in the canonical (NVT) ensemble using Nosé−Hoover thermostats.[6] The time step was set as 1 fs with a time period of 5 ps.

The adsorption energies per atom (E_{ads}) for TM_6 clusters supported on C_2N are calculated by:

$$E_{ads} = \frac{E_{TM_6@C_2N} - \left(E_{C_2N} + E_{TM_6}\right)}{6} \quad (1)$$

Where $E_{TM_6@C_2N}$, E_{C_2N}, are the total energy of TM_6@C_2N and C_2N respectively.

E_{TM_6} is the total energy of gas-phase TM_6.

The cohesion energy per atom (E_{coh}) for TM_6 clusters supported on C_2N are calculated by:

$$E_{coh} = \frac{E_{TM_6} - nE_{TM_1}}{6} \quad (2)$$

E_{TM_1} is the total energy of a single TM atom in gas phase.

The typical reaction pathways for OER and ORR on C_2N supported clusters and single-atom catalysts were calculated according to electrochemical framework reported by Nørskov et al.[7] We considered the implicit water solvent model with VASPsol in the calculations.[8] For OER under an acidic reaction environment, the elementary reaction steps can be expressed as:

$$* + H_2O \rightarrow {}^*OH + H^+ + e^- \quad (3)$$

$${}^*OH \rightarrow {}^*O + H^+ + e^- \quad (4)$$

$${}^*O + H_2O \rightarrow {}^*OOH + H^+ + e^- \quad (5)$$

$${}^*OOH \rightarrow {}^* + O_2 + H^+ + e^- \quad (6)$$

The ORR via the 4e⁻ reaction pathway in an acidic electrolyte through elementary steps takes the reverse direction of OER:

$$* + O_2 + H^+ + e^- \rightarrow {}^*OOH \quad (7)$$

$${}^*OOH + H^+ + e^- \rightarrow {}^*O + H_2O \quad (8)$$

$${}^*O + H^+ + e^- \rightarrow {}^*OH \quad (9)$$

$${}^*OH + H^+ + e^- \rightarrow * + H_2O \quad (10)$$

The Gibbs free energy of the intermediate is calculated by:

$$G = E + E_{ZPE} - T \times S \quad (11)$$

where the E is the total energy calculated by DFT calculations, E_{ZPE} is zero point energy and S is the entropy at 298.15 K.

According to equations (1) - (4), the reaction free energy change can be obtained by:

$$\Delta G_1 = G_{*OH} + \frac{1}{2}G_{H_2} - G_* - G_{H_2O} \quad (12)$$

$$\Delta G_2 = G_{*O} + \frac{1}{2}G_{H_2} - G_{*OH} \quad (13)$$

$$\Delta G_3 = G_{*OOH} + \frac{1}{2}G_{H_2} - G_{*O} - G_{H_2O} \quad (14)$$

$$\Delta G_4 = 4.92 - \Delta G_{*OOH} \quad (15)$$

The OER and ORR overpotentials can be calculated using the following formulas:

$$\eta^{OER} = \frac{max(\Delta G_1, \Delta G_2, \Delta G_3, \Delta G_4)}{e} - 1.23 \quad (16)$$

$$\eta^{ORR} = 1.23 - \frac{min(\Delta G_1,\ \Delta G_2,\ \Delta G_3,\ \Delta G_4)}{e} \quad (17)$$

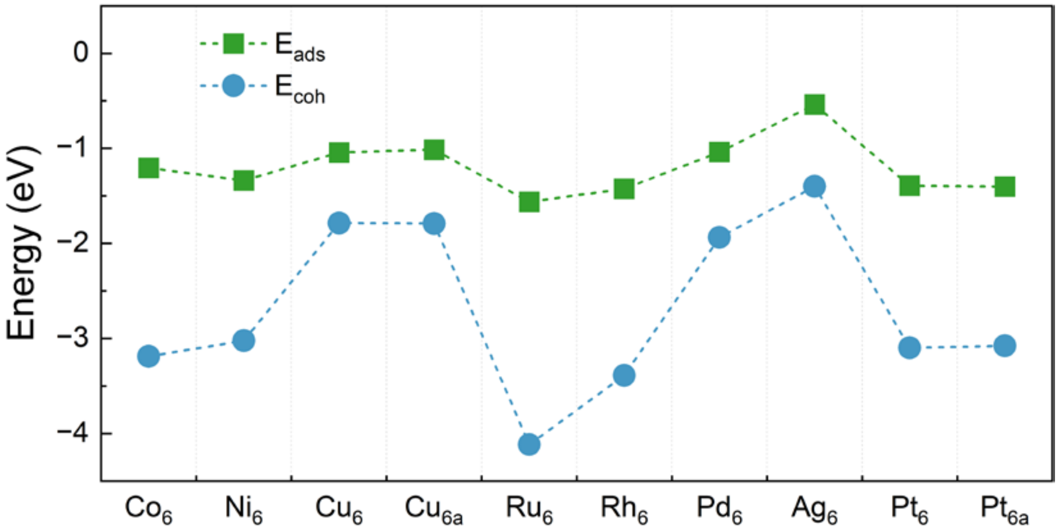

Figure S1. The adsorption energies and cohesive energies of TM_6 clusters supported on C_2N.

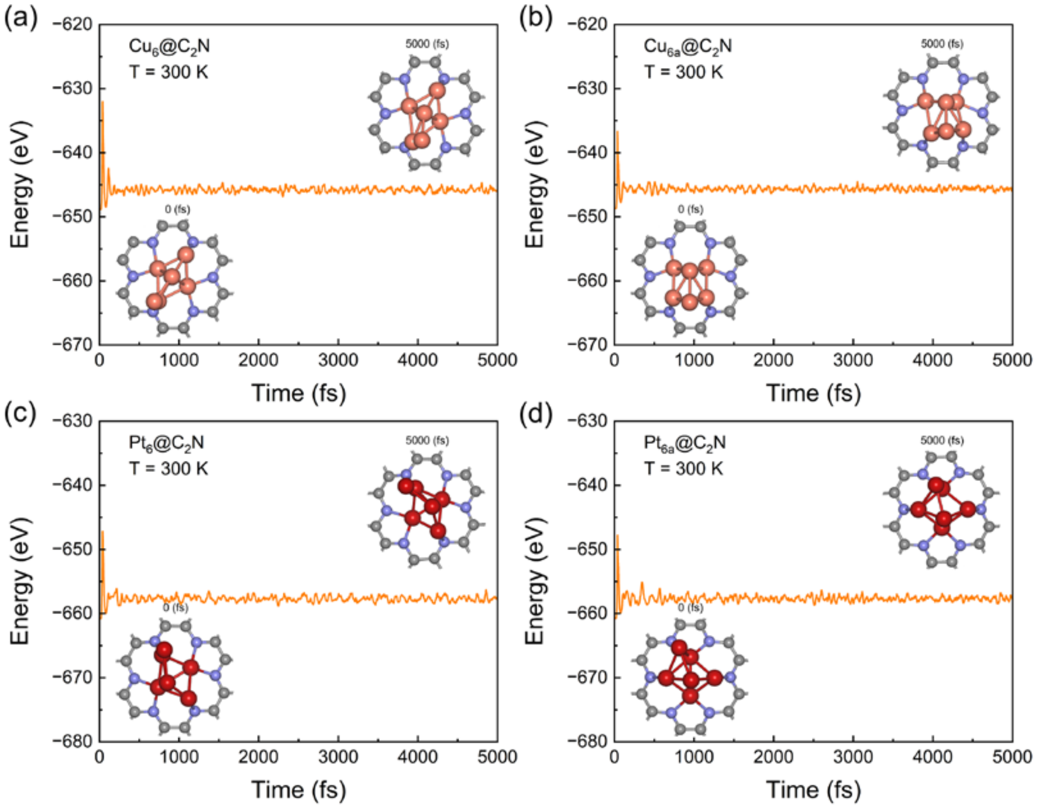

Figure S2. AIMD simulations of (a) Cu_6@C_2N, (b) Cu_{6a}@C_2N, (c) Pt_6@C_2N, and (d) Pt_{6a}@C_2N.

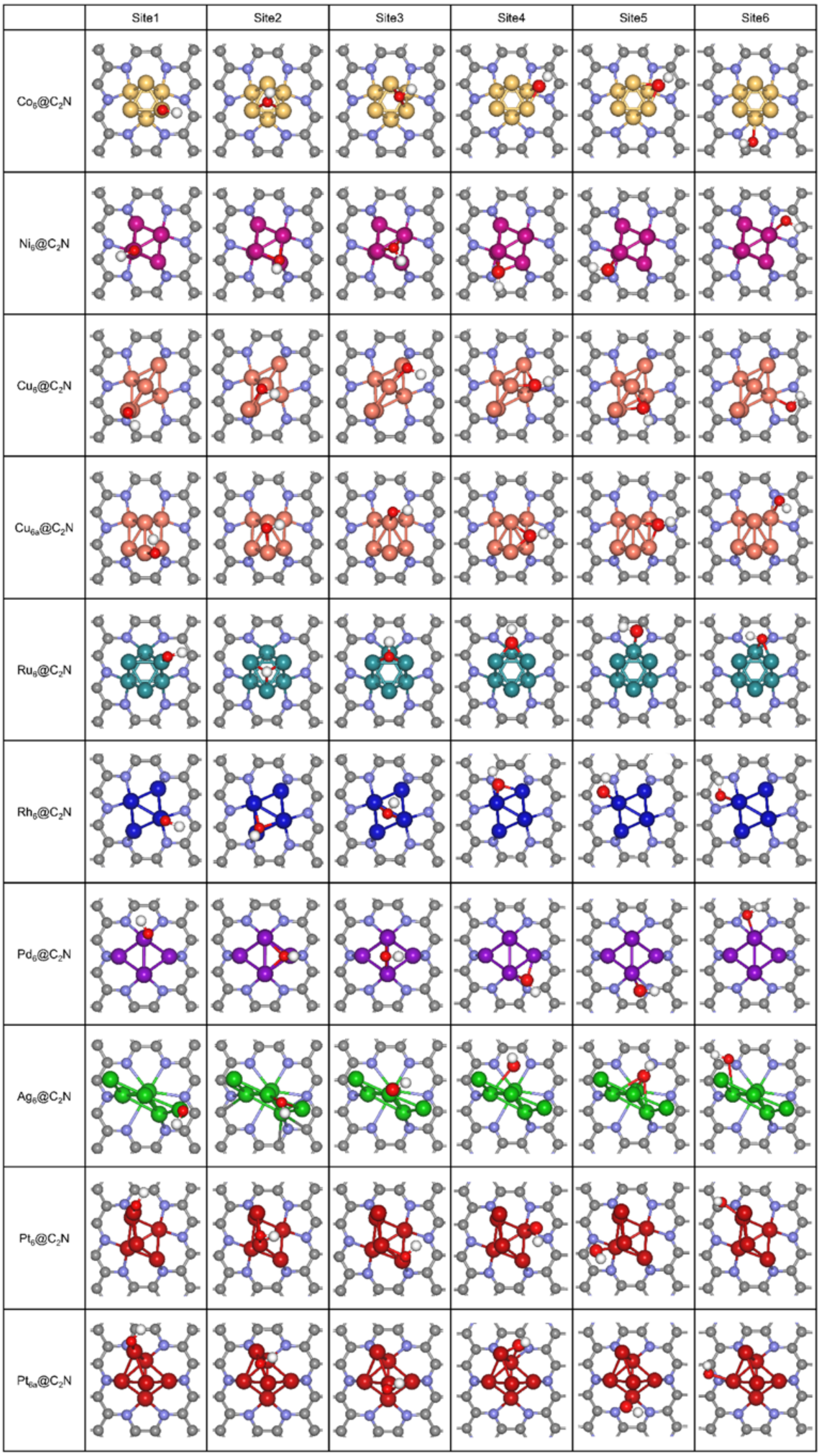

Figure S3. The structure of OH adsorbs on typical sites of TM_6 clusters supported on C_2N.

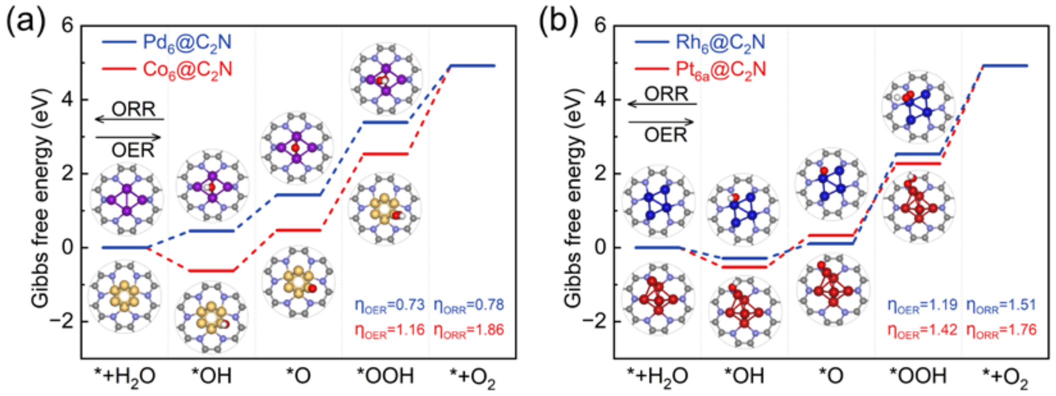

Figure S4. The free energy diagrams of ORR and OER on (a) Pd_6@C_2N and Co_6@C_2N, (b) Rh_6@C_2N and Pt_{6a}@C_2N.

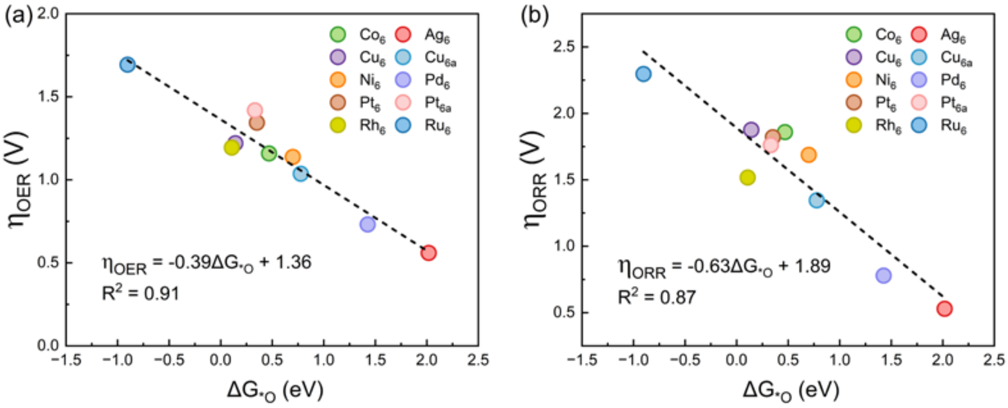

Figure S5. (a) The correlation between the adsorption free energies of O species and OER overpotentials of TM_6@C_2N, (b) The correlation between the adsorption free energies of O species and ORR overpotentials of TM_6@C_2N.

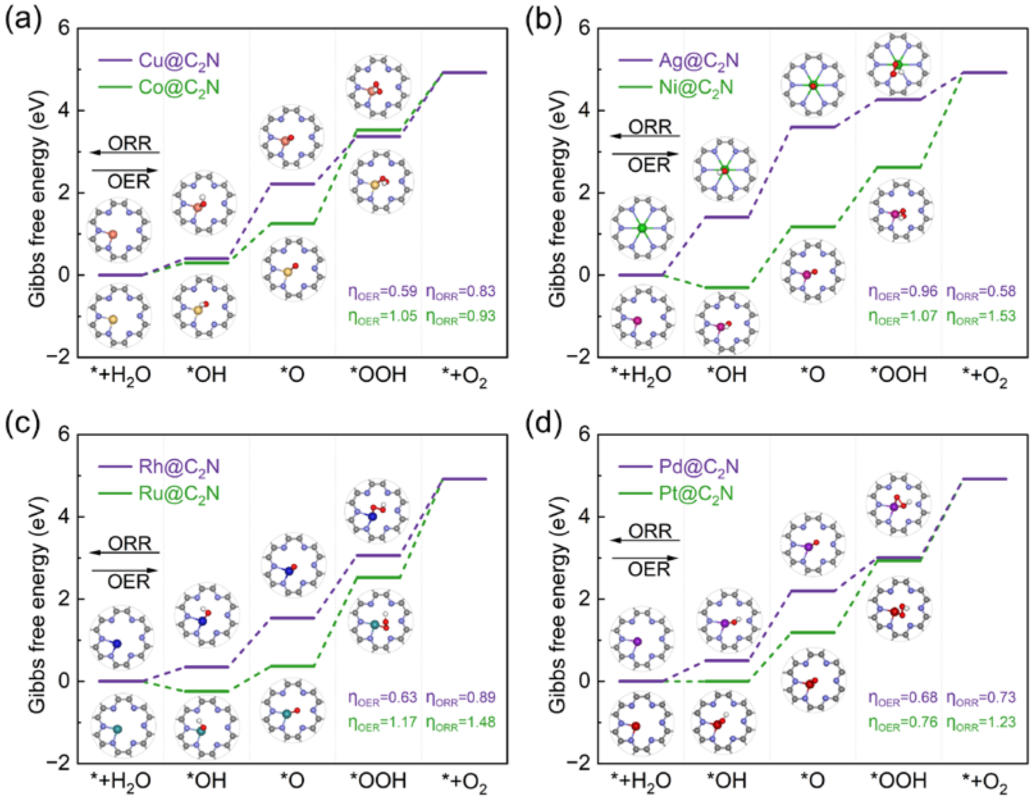

Figure S6. The free energy diagrams on C_2N supported SACs (a) Cu@C_2N and Co@C_2N, (b) Ag@C_2N and Ni@C_2N, (c) Rh@C_2N and Ru@C_2N, (d) Pd@C_2N and Pt@C_2N.

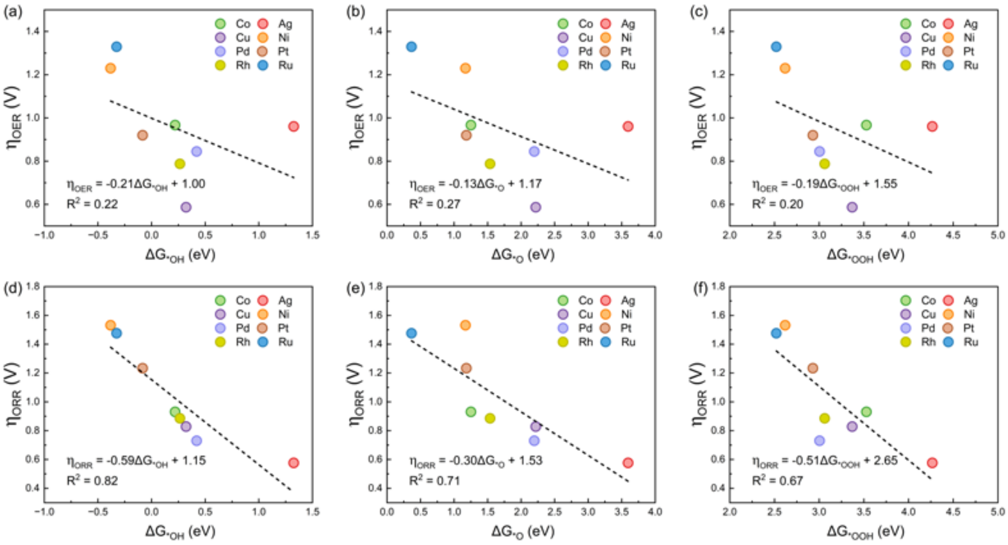

Figure S7. The correlations between the overpotentials of OER and ORR on SACs with adsorption free energies of intermediates: (a) η_{OER} vs. ΔG_{*OH}, (b) η_{OER} vs. ΔG_{*O}. (c) η_{OER} vs. ΔG_{*OOH}, (d) η_{ORR} vs. ΔG_{*OH}, (e) η_{ORR} vs. ΔG_{*O}. (f) η_{ORR} vs. ΔG_{*OOH}.

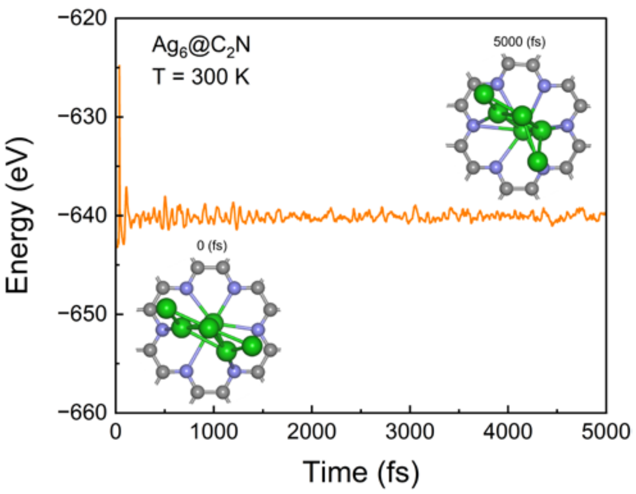

Figure S8. AIMD simulations of $Ag_6@C_2N$ at reaction temperature (300K).

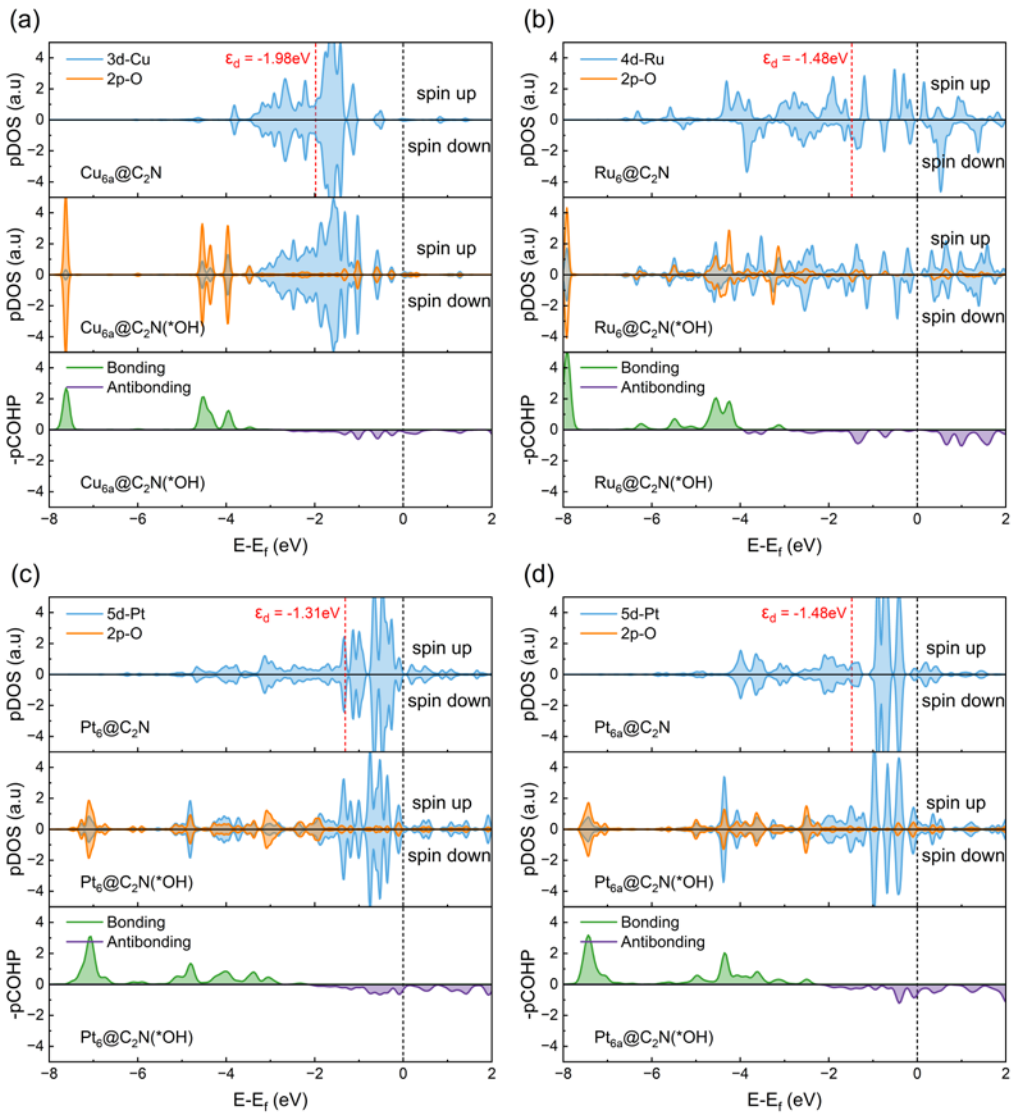

Figure S9. Projected density of states (PDOS) for pristine and OH adsorbed cluster as well as projected crystal orbital Hamilton population (pCOHP) for OH adsorbed on (a) $Cu_{6a}@C_2N$, (b)

Ru_6@C_2N, (c) Pt_6@C_2N and (d) Pt_{6a}@C_2N. The Fermi energy level is set to 0 eV. Note that the ICOHP is calculated based on the interaction between reaction intermediate and TM atoms in adsorption site.

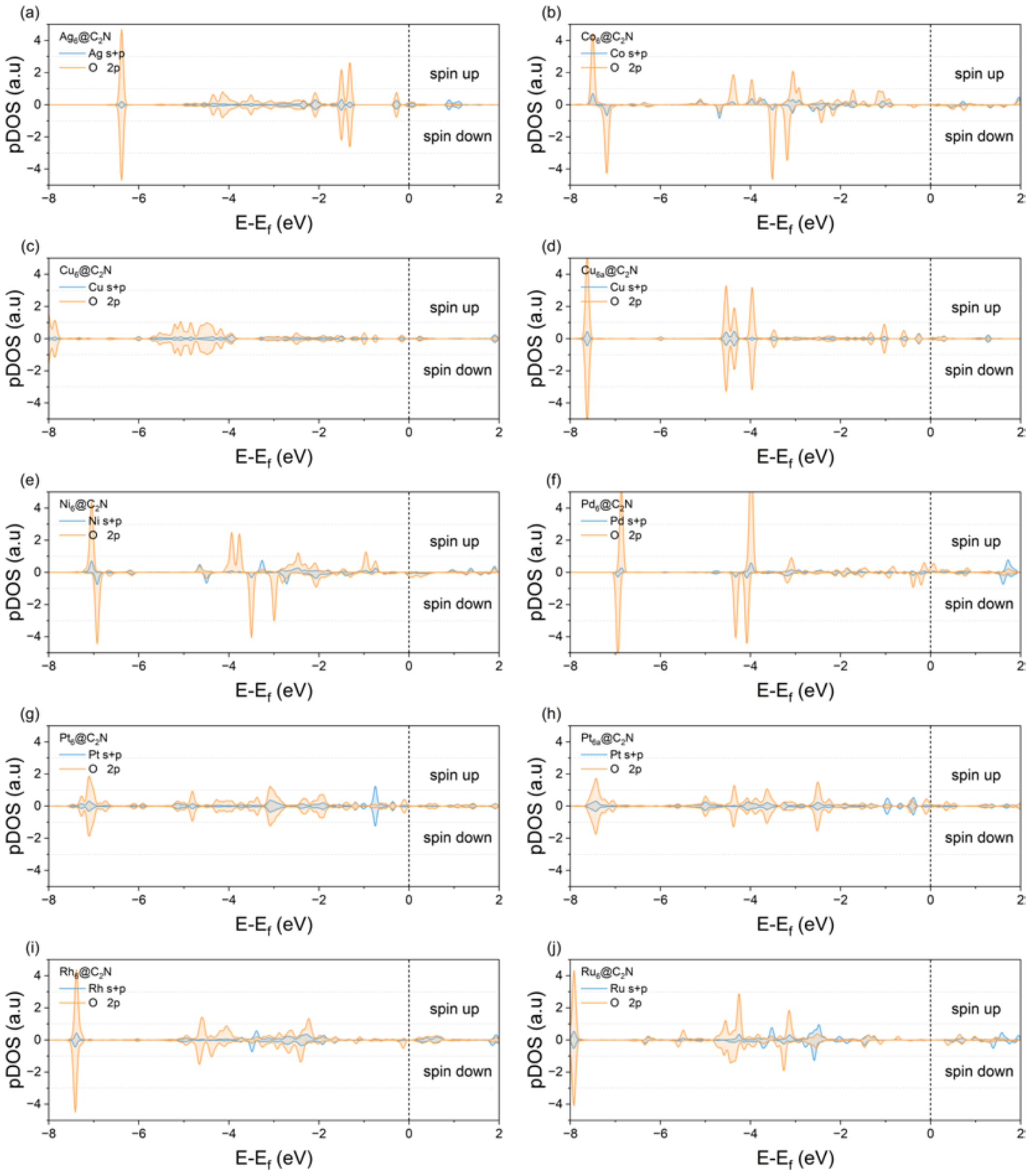

Figure S10. PDOS of sp states for OH adsorbed cluster (a) Ag_6@C_2N, (b) Co_6@C_2N, (c) Cu_6@C_2N, (d) Cu_{6a}@C_2N, (e) Ni_6@C_2N, (f) Pd_6@C_2N, (g) Pt_6@C_2N, (h) Pt_{6a}@C_2N, (i) Rh_6@C_2N and (j) Ru_6@C_2N. The Fermi energy level is set to 0 eV.

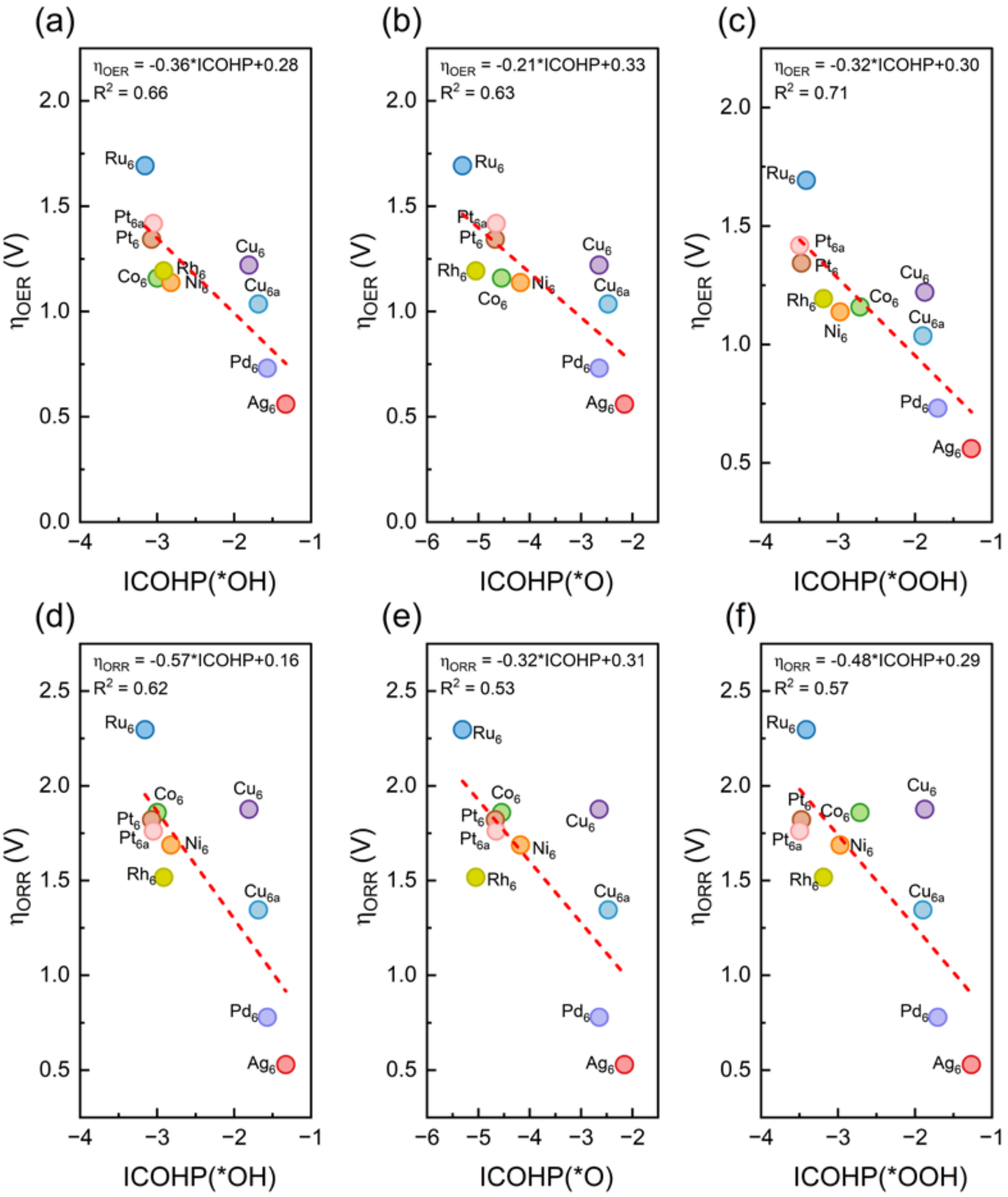

Figure S11. (a-c) correlation between OER overpotential of $TM_6@C_2N$ and ICOHP(*OH), ICOHP(*O), ICOHP(*OOH) respectively; (d-f) correlation between ORR overpotential of $TM_6@C_2N$ and ICOHP(*OH), ICOHP(*O), ICOHP(*OOH) respectively.

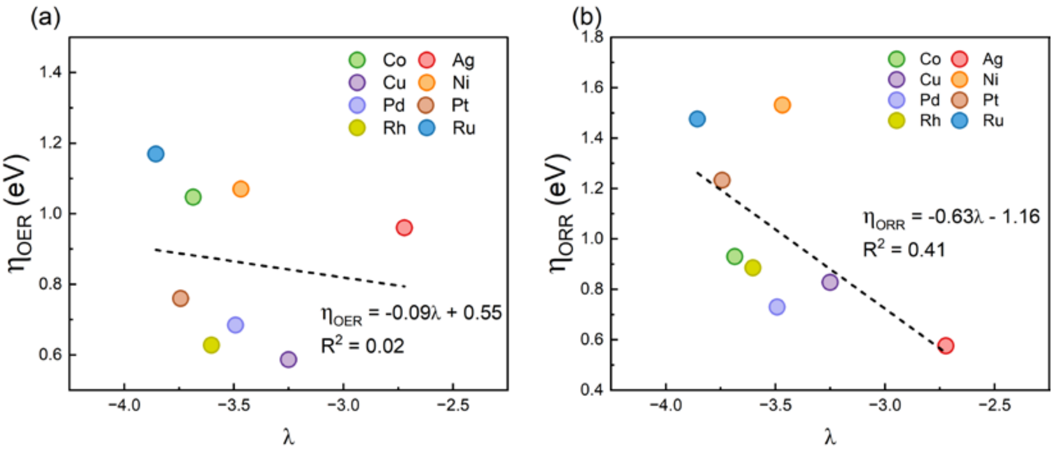

Figure S12. The correlation between the descriptor (λ = $ICOHP_{(OH\text{-}TM)}\text{-}N_{TM}$) and overpotentials of SACs (a) OER and (b) ORR.

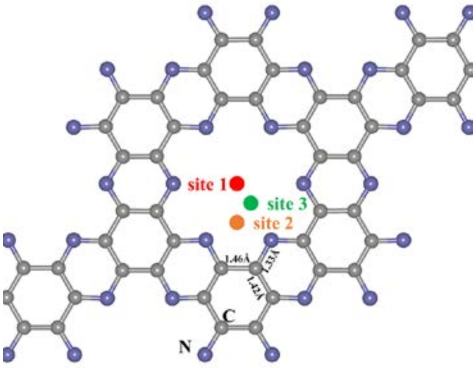

Figure S13. The structure of pristine C_2N (the gray and blue atoms represent carbon and nitrogen atoms, respectively).

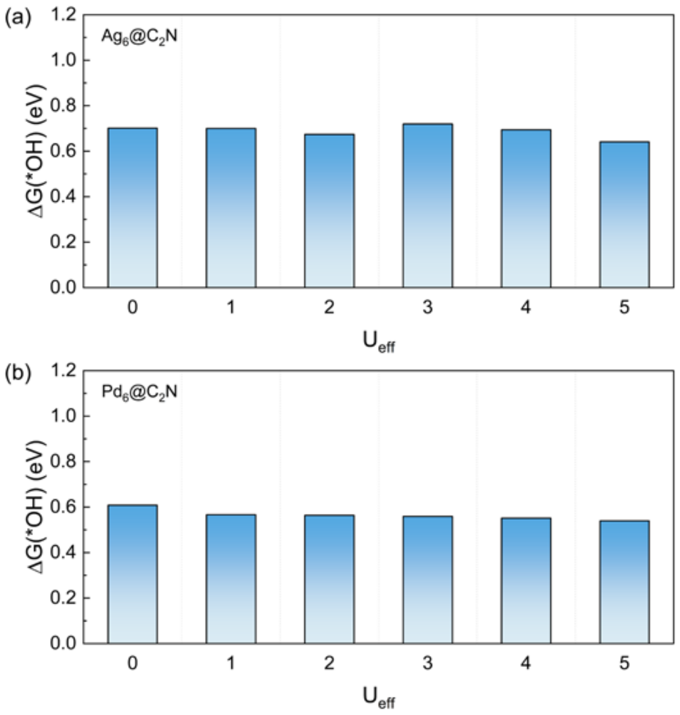

Figure S14. The adsorption energy of OH calculated with various U values for two typical cluster catalysts: (a) $Ag_6@C_2N$ and (b) $Pd_6@C_2N$.

Table S1. E_{TM_6} is the total energy of gas-phase TM_6. E_{TM_1} is the total energy of gas-phase TM_1. $E_{TM_6@C_2N}$ is the total energy of $TM_6@C_2N$. The adsorption energies per atom (E_{ads}). The cohesive energy per atom (E_{coh}). (Unit: eV)

**$TM_6@C_2N$**	E_{TM_6}	E_{TM_1}	$E_{TM_6@C_2N}$	E_{ads}	E_{coh}
$Co_6@C_2N$	-29.08	-1.66	-667.37	-1.21	-3.19
$Ni_6@C_2N$	-19.84	-0.29	-658.93	-1.34	-3.02
$Cu_6@C_2N$	-12.17	-0.24	-649.48	-1.04	-1.79
$Cu_{6a}@C_2N$	-12.20	-0.24	-649.33	-1.01	-1.79
$Ru_6@C_2N$	-36.90	-2.03	-677.32	-1.56	-4.12
$Rh_6@C_2N$	-28.16	-1.30	-667.76	-1.42	-3.39
$Pd_6@C_2N$	-20.46	-1.47	-657.75	-1.04	-1.94
$Ag_6@C_2N$	-9.58	-0.20	-643.88	-0.54	-1.40
$Pt_6@C_2N$	-22.04	-0.58	-661.45	-1.39	-3.10
$Pt_{6a}@C_2N$	-21.92	-0.58	-661.39	-1.40	-3.08

Table S2. Adsorption free energies (eV) for OH adsorbs on typical sites of $TM_6@C_2N$. (Unit: eV)

**$TM_6@C_2N$**	**ΔG(site1)**	**ΔG(site2)**	**ΔG(site3)**	**ΔG(site4)**	**ΔG(site5)**	**ΔG(site6)**
$Co_6@C_2N$	-0.63	-0.13	1.41	1.22	0.05	1.19
$Ni_6@C_2N$	-0.46	0.04	-0.37	0.85	0.26	0.49
$Cu_6@C_2N$	0.10	-0.17	-0.65	0.25	1.14	1.45
$Cu_{6a}@C_2N$	-0.01	-0.11	0.11	0.21	0.40	0.59
$Ru_6@C_2N$	-1.07	0.89	0.84	0.37	0.22	0.26
$Rh_6@C_2N$	-0.29	0.29	-0.02	0.73	0.68	1.12
$Pd_6@C_2N$	0.51	0.75	0.45	1.75	1.65	1.33
$Ag_6@C_2N$	0.74	0.70	0.89	1.93	1.80	2.51
$Pt_6@C_2N$	-0.59	-0.28	-0.26	1.83	1.79	1.39
$Pt_{6a}@C_2N$	-0.53	-0.20	-0.11	0.45	1.88	0.76

Table S3. Adsorption free energy (eV) for *OH, *O, and *OOH on the $TM_6@C_2N$ and $TM@C_2N$, and the corresponding OER and ORR overpotentials (V).

**Models**	**ΔG_{*OH}**	**ΔG_{*O}**	**ΔG_{*OOH}**	η_{OER}	η_{ORR}
$Co_6@C_2N$	-0.63	0.47	2.53	1.16	1.86
$Ni_6@C_2N$	-0.46	0.70	3.07	1.14	1.69
$Cu_6@C_2N$	-0.65	0.14	2.59	1.22	1.88
$Cu_{6a}@C_2N$	-0.11	0.78	3.04	1.04	1.34
$Ru_6@C_2N$	-1.07	-0.90	2.02	1.69	2.30
$Rh_6@C_2N$	-0.29	0.11	2.53	1.19	1.52
$Pd_6@C_2N$	0.45	1.43	3.39	0.73	0.78
$Ag_6@C_2N$	0.70	2.02	3.81	0.56	0.53
$Pt_6@C_2N$	-0.59	0.35	2.35	1.34	1.82
$Pt_{6a}@C_2N$	-0.53	0.33	2.27	1.42	1.76
$Co@C_2N$	0.30	1.25	3.53	1.05	0.93
$Ni@C_2N$	-0.30	1.17	2.62	1.07	1.53
$Cu@C_2N$	0.40	2.22	3.37	0.59	0.83
$Ru@C_2N$	-0.25	0.37	2.52	1.17	1.48
$Rh@C_2N$	0.34	1.54	3.06	0.63	0.89
$Pd@C_2N$	0.50	2.20	3.01	0.68	0.73
$Ag@C_2N$	1.41	3.60	4.27	0.96	0.58
$Pt@C_2N$	0.00	1.18	2.93	0.76	1.23

Table S4. Integrated crystal orbital Hamilton populations (ICOHP) of *O, *OH, *OOH and the number of transition metal atoms in adsorption site (N_{TM}).

**Models**	**$ICOHP_{(OH-TM)}$**	**$ICOHP_{(O-TM)}$**	**$ICOHP_{(OOH-TM)}$**	**N_{TM}**
$Co_6@C_2N$	-3.00	-4.55	-2.71	1
$Ni_6@C_2N$	-2.82	-4.18	-2.97	1
$Cu_6@C_2N$	-1.81	-2.65	-1.87	2
$Cu_{6a}@C_2N$	-1.68	-2.47	-1.90	2
$Ru_6@C_2N$	-3.16	-5.31	-3.41	1
$Rh_6@C_2N$	-2.91	-5.05	-3.19	1
$Pd_6@C_2N$	-1.57	-2.65	-1.70	2
$Ag_6@C_2N$	-1.33	-2.15	-1.27	2
$Pt_6@C_2N$	-3.07	-4.67	-3.47	1
$Pt_{6a}@C_2N$	-3.05	-4.65	-3.50	1

References:

(1) Blöchl, P. E. Projector augmented-wave method. *Phys. Rev. B.* **1994**, *50*, 17953.

(2) Kresse, G.; Furthmüller, J. Efficient iterative schemes for ab initio total-energy calculations using a plane-wave basis set. *Phys. Rev. B.* **1996**, *54*, 11169.

(3) Kresse, G.; Hafner, J. Ab initio molecular dynamics for liquid metals. *Phys. Rev. B.* **1993**, *47*, 558.

(4) Perdew, J. P.; Burke, K.; Ernzerhof, M. Generalized gradient approximation made simple. *Phys. Rev. Lett.* **1996**, *77*, 3865.

(5) Fabris, S.; de Gironcoli, S.; Baroni, S.; Vicario, G.; Balducci, G. Taming multiple valency with density functionals: A case study of defective ceria. *Phys. Rev. B.* **2005**, *71*, 041102.

(6) Hoover, W. G. Canonical dynamics: Equilibrium phase-space distributions. *Phys. Rev. A.* **1985**, *31*, 1695.

(7) Nørskov, J. K.; Rossmeisl, J.; Logadottir, A.; Lindqvist, L.; Kitchin, J. R.; Bligaard, T.; Jonsson, H. Origin of the overpotential for oxygen reduction at a fuel-cell cathode. *J. Phys. Chem. B.* **2004**, *108*, 17886-17892.

(8) Mathew, K.; Sundararaman, R.; Letchworth-Weaver, K.; Arias, T.; Hennig, R. G. Implicit solvation model for density-functional study of nanocrystal surfaces and reaction pathways. *J. Phys. Chem. C.* **2014**, *140*.
